# Complete genome sequence of dengue virus serotype 2 obtained from Chattogram, Bangladesh

**DOI:** 10.1128/mra.00023-25

**Published:** 2025-04-17

**Authors:** Mohabbat Hossain, Md. Abdur Rob, M. A. Sattar, Abul Faisal Md. Nuruddin Chowdhury, Noor Mohammed, Istiaq Uddin Ahmed, Mohammed Maruf ul Quader, H. M. Hamidullah Mehedi, Md. Zakir Hossain, Md. Mobarok Hossain, Md. Mustafizur Rahman, Md. Shaheen Alam, Mohammad Jubair, Anjasu Paul, Susmita Barua, Md. Mahbub Hasan, Afroza Akter Tanni, S. M. Rafiqul Islam, Adnan Mannan

**Affiliations:** 1 Department of Genetic Engineering and Biotechnology, Faculty of Biological Sciences, University of Chittagong 54493, Chattogram, Bangladesh; 2 Department of Medicine, Chittagong Medical College 467859, Chattogram, Bangladesh; 3 Asperia Health Research and Development Foundation 622458, Chattogram, Bangladesh; 4 Department of Paediatric Nephrology, Chittagong Medical College 467859, Chattogram, Bangladesh; 5 Department of Medicine, 250 Bedded General Hospital Chattogram 467859, Chattogram, Bangladesh; 6 Department of Microbiology, Bangladesh Institute of Tropical & Infectious Diseases (BITID) 467859, Chattogram, Bangladesh; 7 Virology Laboratory, Infectious Diseases Division, Icddr,b 56291, Mohakhali, Dhaka, Bangladesh; 8 Next Generation Sequencing, Research and Innovation Laboratory Chittagong (NRICh), Biotechnology Research & Innovation Centre (BRIC), University of Chittagong 54493, Chattogram, Bangladesh; DOE Joint Genome Institute, Berkeley, California, USA

**Keywords:** Dengue, Bangladesh, Chattogram, WGS, DENV-2

## Abstract

The whole genome sequence of a dengue virus serotype 2 strain (GenBank accession number PQ657766) obtained from a dengue-infected hospitalized patient in Chittagong, Bangladesh, is classified as the cosmopolitan genotype V, with genetic alterations observed in several structural proteins. The genomic data were generated using Oxford Nanopore sequencing technology.

## ANNOUNCEMENT

Dengue virus (DENV), an *Orthoflavivirus* in the *Flaviviridae* family*,* is a mosquito-borne virus that causes dengue fever and has an ~10.7 kb positive-sense RNA genome. In rare cases, infection progresses to dengue hemorrhagic fever or fatal dengue shock syndrome (1–3). Bangladesh’s 2023 dengue outbreak was the deadliest, with 1,705 deaths (4), while over 14 million dengue cases and more than 10,000 deaths have been recorded globally in 2024 (5). Enhanced genomic surveillance is crucial to track DENV lineages (6) and aid preventive measures (7).

Blood serum samples were collected from dengue patients in government tertiary hospitals in Chittagong during the 2023 outbreak. Viral RNA was extracted using Chemagic Viral NA/gDNA H96 Kit (CMG-1049) on the Chemagic 360 system (PerkinElmer, USA) following the manufacturer’s instructions. A real-time RT-PCR assay using the TaqPath 1-Step Multiplex Master Mix (No ROX) Kit (Thermo Fisher Scientific, USA) was performed on the Bio-Rad CFX96 Touch real-time PCR system (Bio-Rad, USA) to determine the DENV serotypes. DENV serotyping primers and probes were sourced from a published article (8). Among the identified DENV serotypes, a DENV-2 positive (severe, hospitalized) case with a Ct value < 25 underwent whole-genome sequencing. cDNA was prepared from the isolated viral RNA using the PrimeScript cDNA Synthesis Kit (Takara Bio Inc., Japan) as per the manufacturer’s protocol, followed by a multiplex PCR that was performed with GoTaq G2 Hot Start Polymerase (Promega Corporation, USA) and specific primer pools (9). Oxford Nanopore sequencing technology was utilized for the whole genome sequencing of the DENV-2 serotype. Sequencing libraries were prepared according to the manufacturer’s V14 Ligation Sequencing protocol (Native Barcoding Kit V14 96, SQK-NBD114.96). The barcoded library (50 fmol) was loaded onto a MinION R10.4.1 Flowcell (FLO-MIN114) and sequenced on the MinION MK 1C platform. Base calling was performed using bcl2fastq, yielding 19.1M bases, with an average read length of 1.1 Kb, assessed by FastQC. Adapters/primers were removed using Trimmomatic software. Minimap2 version 2.28 (https://github.com/lh3/minimap2) was used for genome mapping against the reference genome NC_001474. The number of reads that were mapped to the reference genome was 10,723 bp, with a coverage depth of 82.5×. The completeness of the sequenced genome was determined by comparing it with the reference genome (NC_001474). The coverage breadth of our sequence was around 99%.

**Fig 1:**
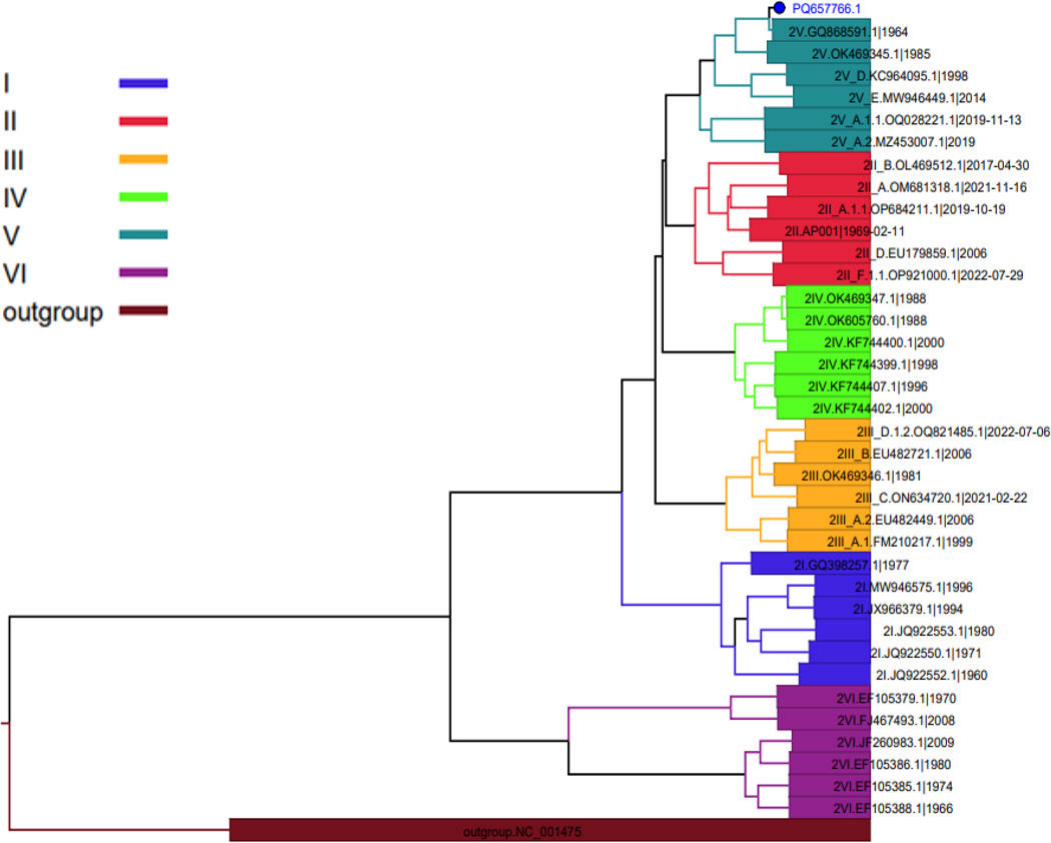
The randomly selected reference sequences were aligned with the study sequence using the Augur pipeline (https://github.com/nextstrain/augur). The tree was built using IQ-TREE 2 (https://github.com/iqtree/iqtree2) with 1,000 bootstraps (maximum likelihood). The analyzed sequence clusters within the DENV-2 serotype were classified into genotype V (cosmopolitan genotype) after comparison with representative global sequences.

The Kraken2 viral database was utilized to obtain a taxonomic identification. Analysis with BLASTn showed that the viral genome exhibited 99.57% similarity with the genomic sequence of the DENV-2 strain from Thailand (GQ868591). Phylogenetic analysis of whole genome sequences placed the Chittagong isolate PQ657766 within DENV-2 genotype-V (Fig. 1), linking it to a Southeast Asian/American lineage found in Southeast Asia and Central/South America. Notable genetic alterations were found in the DENV-2 serostructural proteins, including polyprotein, anchored capsid protein, membrane glycoprotein precursor, and membrane glycoprotein (Table 1).

**TABLE 1:** List of genes in the analyzed DENV-2 sequence, including amino acid alterations in structural proteins (accession number of the reference genome: NC_001474)

Protein name	Gene name	Amino acid changes	List of alterations in the protein sequence
Structural protein	Polyprotein (POLY)	12	K9R (122A > G), S101T (397T > A 399T > C), M104I (408G > A), L108M (418C > A), D143N (523G > A), V234A (797T > C 798C > A), I241V (817A > G), M249I (843G > A), H262Y (880C > T), A266I (892G > A 893C > T), T276A (922A > G), M286I (954G > A)
	Anchored capsid protein (ancC)	4	K9R (122A > G), S101T (397T > A 399T > C), M104I (408G > A), L108M (418C > A)
	Capsid protein (C)	1	K9R (122A > G)
	Membrane glycoprotein precursor (prM)	7	D29N (523G > A), V120A (797T > C 798C > A), I127V (817A > G), M135I (843G > A), H148Y (880C > T), A152I (892G > A 893C > T), T162A (922A > G)
	Protein (pr)	1	D29N (523G > A)
	Membrane glycoprotein (M)	6	V29A (797T > C 798C > A), I36V (817A > G), M44I (843G > A), H57Y (880C > T), A61I (892G > A 893C > T), T71A (922A > G)
	Envelope protein (E)	1	M6I (954G > A)

## Data Availability

The assembled genome was deposited in GenBank under accession number PQ657766, and the reads were submitted under SRA ID SRR32137766.
